# Associations of muscle and adipose tissue parameters with long-term outcomes in middle and low rectal cancer: a retrospective cohort study

**DOI:** 10.1186/s40644-022-00514-x

**Published:** 2023-01-12

**Authors:** Jiyang Liu, Xiongfeng Yu, Xueqing Huang, Qingquan Lai, Jieyun Chen

**Affiliations:** 1 Department of Radiology, Quanzhou First Hospital Affiliated to Fujian Medical University, 248-252 N, Dong Street, Quanzhou City, Fujian Province China; 2 Department of Radiology, Second Affiliated Hospital of Fujian Medical University, 34 N, Zhong-Shan-Bei Street, Quanzhou City, Fujian Province China

**Keywords:** Body composition analysis, Non-enhanced CT imaging, Rectal cancer, Prognosis

## Abstract

**Objective:**

To investigate the role of preoperative body composition analysis for muscle and adipose tissue distribution on long-term oncological outcomes in patients with middle and low rectal cancer (RC) who received curative intent surgery.

**Methods:**

A total of 155 patients with middle and low rectal cancer who underwent curative intent surgery between January 2014 and December 2016 were included for the final analysis. Skeletal muscle area (SMA), skeletal muscle radiodensity (SMD), visceral fat area (VFA) and mesorectal fat area (MFA) were retrospectively measured using preoperative CT images. To standardize the area according to patient stature, SMA was divided by the square of the height (m^2^) and the skeletal muscle mass index (SMI, cm^2^/m^2^) was obtained. Each median values of the distribution in male and female served as cut-off point for SMI, SMD, VFA, and MFA, respectively. Univariate and multivariate analysis were performed to evaluate the association between body composition and long-term oncological outcomes. Overall survival (OS) measured in months from the day of primary surgery until death for any cause. Disease-free survival (DFS) was defined as the interval between surgery and tumor recurrence. The Kaplan-Meier method with log-rank testing was used to validate prognostic biomarkers. Intraclass correlation coefficient (ICC) was used to evaluate interobserver and intraobserver reproducibility for SMA, SMD, MFA,VFA.

**Results:**

During the follow-up period, 42 (27.1%) patients had tumor recurrence; 21 (13.5%) patients died. The sex-specific median value of SMI was 28.6 cm^2^/m^2^ for females and 48.2 cm^2^/m^2^ for males. The sex-specific median value of SMD was 34.7 HU for females and 37.4 HU for males. The sex-specific median value of VFA was 123.1 cm^2^ for females and 123.2 cm^2^ for males. The sex-specific median value of MFA was 13.8 cm^2^ for females and 16.0 cm^2^ for males. In the Cox regression multivariate analysis, SMI (*P* = 0.036), SMD (*P* = 0.022), and postoperative complications grades (*P* = 0.042) were significantly different between death group and non-death group; SMD (*P* = 0.011) and MFA (*P* = 0.022) were significantly different between recurrence group and non-recurrence group. VFA did not show any significant differences. By the Kaplan-Meier method with log-rank testing, DFS was significantly longer in patients with high-MFA (*P* = 0.028) and shorter in patients with low-SMD (*P* = 0.010), OS was significantly shorter in patients with low-SMI (*P* = 0.034) and low-SMD (*P* = 0.029).

**Conclusions:**

Quantitative evaluation of skeletal muscle mass and adipose tissue distributions at initial diagnosis were important predictors for long-term oncologic outcomes in RC patients. SMD and SMI were independent factors for predicting OS in patients with middle and low rectal cancer who had radical surgery. SMD and MFA were independent factors for predicting DFS in patients with middle and low rectal cancer who had radical surgery.

## Introduction

According to the latest statistics from American Cancer Society (ASC), colorectal cancer (CRC) is the second leading cause of cancer-related deaths in the United States with 44,850 estimated new cases of rectal cancer (RC) in 2022 [1]. Identification of reliable and potentially modifiable factors associated with long-term oncologic outcomes is clinically important for vulnerable cancer patients. Body composition has gained increasing attention in oncology in recent years due to fact that quantitative evaluation of skeletal muscle mass and adipose tissue distributions has been revealed to offer prognostic implications for many different cancer types, including RC [2–4].

Sarcopenia, indicated by both loss of muscle mass (myopenia) and declining muscle function, is categorized as primary when age-associated, or secondary due to pathogenic mechanisms [5]. In some previous studies, sarcopenia was demonstrated to associate with poor outcomes in RC patients [6, 7]. Obesity has been reported as a potential risk factor for adverse clinical outcomes in patients with RC [8]. Body mass index (BMI), as an incomplete and crude indicator of total body fat, is widely used in obesity-related research, although it hardly reflects body fat distribution [9]. There is increasing evidence that visceral fat is to be favored over BMI in metabolic syndrome and cancer development [10, 11].

Computed tomography (CT) has emerged as an important tool for accurate body composition analysis of muscle and adipose tissue, because CT scan is a routine part of diagnosis and surveillance in cancers-related diseases [12]. Cross-sectional area of skeletal muscle (SMA) and visceral fat (VFA) from a single axial CT slice at the third lumbar vertebra (L3) were significantly correlated with total body skeletal muscle volume and visceral fat volume, respectively [3]. Previous studies evaluating changes in body composition and its results on RC, had mainly focused on VFA and SMA rather than the amount of mesorectal fat around rectum and the quality of skeletal muscle for oncological outcomes [4, 8, 10, 13]. Yamaoka et al. suggested that the mesorectal fat area (MFA) measured at the level of the tip of sciatic spine is a reliable predictor of whole mesorectal fat volume, while VFA measured at L3 level only represents the size of retroperitoneal, omental, mesenteric, and mesocolic adipose tissue [14]. Total mesorectal excision (TME), resection for the rectum and mesorectal fat within the mesorectal fascia, is the gold standard procedure for RC [15]. Perirectal fat, which represents adipose tissue accumulation around rectum, is a vital anatomical landmark for TME [15]. Thus, MFA perhaps has greater prognostic impacts for RC patients compared with VFA. To better define sarcopenia, the function or strength of skeletal muscle must be taken into consideration. Myosteatosis, defined as an increased infiltration by inter- and intramuscular adipose tissue, has been reported to associate with poor skeletal muscle quality, reflecting the performances of skeletal muscle function [16, 17]. Skeletal muscle radiodensity (SMD), defined as the radiodensity of skeletal muscle measured by CT, is highly correlated with direct measurement of muscle lipid content by biopsy [18]. Thus, Chan et al. suggested that myosteatosis and low SMD refer to the same physiological changes of skeletal muscle [19].

In our study, we aimed to evaluate the association between preoperative CT-based measurements of SMA (normalized for height), SMD, VFA, MFA, and the long-term clinical outcomes of middle and low RC patients who underwent curative intent surgery.

## Materials and methods

This retrospective cohort study was approved by our institutional review board, and written informed consent was waived because of the retrospective nature of the study.

We present the following article in accordance with the STROBE (Strengthening the Reporting of Observational Studies in Epidemiology) reporting checklist.

### Study population

**Fig. 1:**
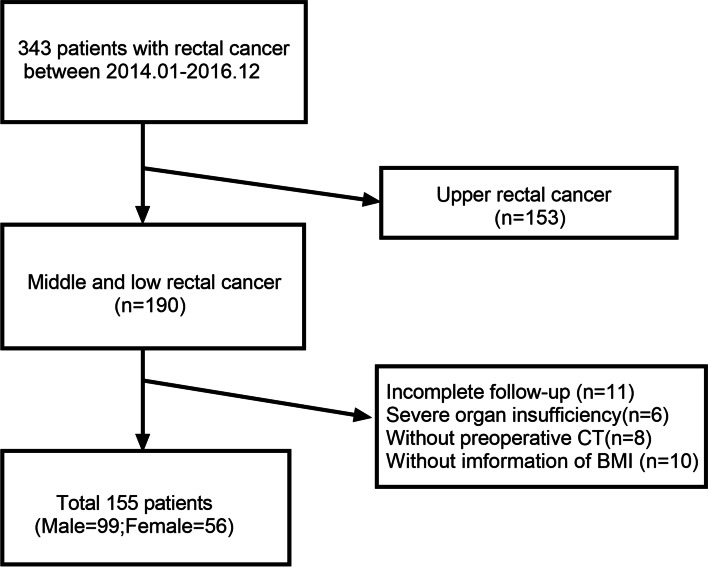
Flowchart of the study

343 consecutive patients with pathologically confirmed RC who underwent laparoscopic radical surgery at our institution between January 2014 and December 2016 were retrospectively analyzed. 153 patients with upper RC (defined as partially peritonalized), 11 patients with incomplete follow-up data, 6 patients with severe organ insufficiency, 8 patients without preoperative CT examinations, 10 patients without information of weight or height, were excluded. A total of 155 patients were enrolled for the statistical analysis. The flowchart of patient selection process was summarized in Fig. 1.

### Demographic and clinical variables

We gathered patient’s sex, diagnosed age, BMI, height, weight, blood carcino-embryonic antigen (CEA), alone with underlying vascular risk history including diabetes, heart disease, hypertension by reviewing electronic medical records. Other baseline characteristics included are American Society of Anesthesiologists score (ASA), hospitalization days, intraoperative blood loss (ml), pathologic TNM tumor stage according to the 7th edition of the American Joint Committee on colorectal cancer staging (AJCC), postoperative complications assessed by the Clavien-Dindo Classification system with 1–2 considered as minor complications and 3 or higher as major complications. Overall survival (OS) measured in months from the day of primary surgery until death for any cause. Disease-free survival (DFS) was defined as the interval between surgery and tumor recurrence, including distant metastasis and local relapse. The electronic medical records system was searched for date of death and general medical information.

### Imaging protocol and body composition measurements

Non-enhanced preoperative CT examinations were conducted on a dual-source CT scanner (SOMATOM Definition, Siemens Healthcare, Forchheim, Germany). Detailed imaging parameters were as following: 120 KVp tube voltage; 150–240 mA tube current with automatic dose modulation; matrix, 512 × 512; collimation, 64 × 0.625 mm; reconstructed slice thickness, 2 mm. Iterative reconstruction algorithm was used for suppressing image noise.

**Fig. 2:**
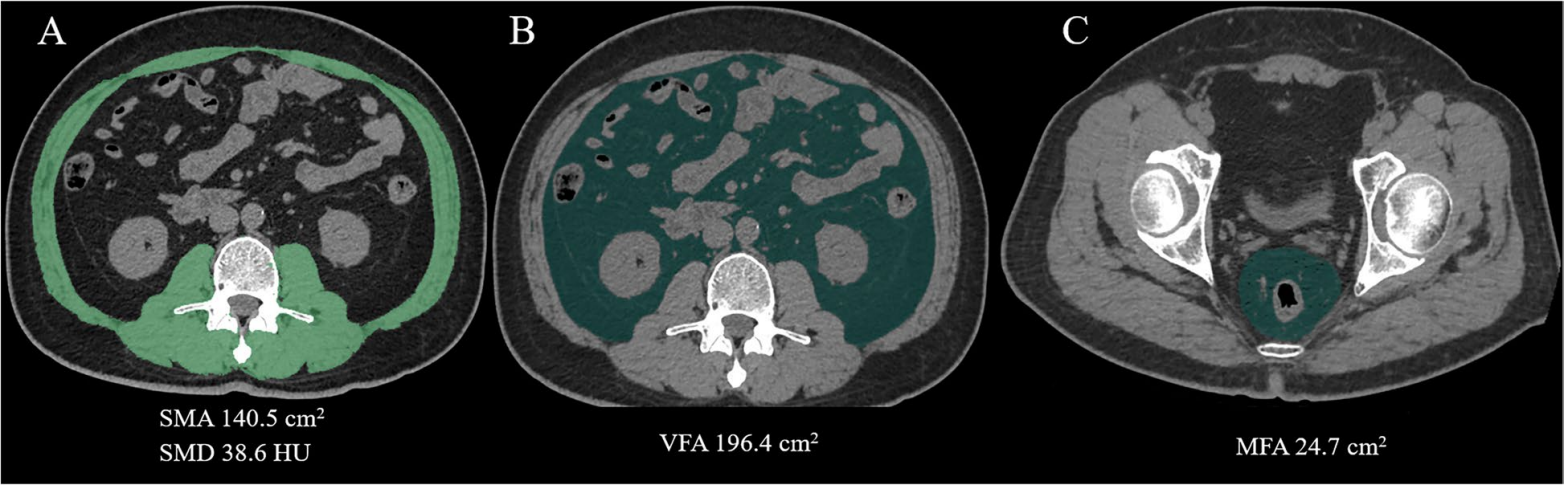
Abdominal axal CT images of patients with quantitative measurements of SMA, SMD, VFA at the level of L3 and MFA at the level of ischial spine

Images were analyzed by using open-source software 3D Slicer 4.11 [20]. At the level of L3, where both transverse processes were visible or most clearly visible in case of scoliosis, SMA was measured by manual correcting all the abdominal wall and back muscles areas obtained from the muscle-density mask (− 30 to 150 HU). To standardize the area according to patient stature, SMA was divided by the square of the height (m^2^) and the skeletal muscle mass index (SMI, cm^2^/m^2^) was obtained. SMD was measured as the mean HU of SMA. From the fat-density mask (− 150 to − 50 HU), VFA was retrieved through manually tracing the abdominal muscular wall at the same L3 level. From the fat-density mask (− 150 to − 50 HU), MFA was obtained by subtracting the rectal area from the mesorectal area at the level of ischial spine with manually tracing. When the outer edge of rectal wall could not be readily visualized in a T3 tumor, we continually traced along the contour of the outer edge of rectal wall [21] (Fig. 2).

In our study, each median values of the distribution in male and female served as cut-off point for SMI, SMD, VFA, and MFA, respectively. Patients were then assigned to either the low- or high-SMI groups, low- or high-SMD groups, low- or high-VFA groups, and low- or high-MFA groups accordingly. Sarcopenia was defined as low-SMI, and myosteatosis was defined as low-SMD.

### Statistical analyses

Statistical analyses were performed with SPSS 25.0 (IBM Corp, Armonk, NY, USA). Differences were considered statistically significant when *p* < 0.05. Data were shown as means with standard deviation (SD) for continuous variables, and frequencies with percentages for categorical variables. Patients were divided into tumor recurrence group and non-recurrence group, as well as death group and non-death group during the 5-year follow-up period. Chi-squared test and Student’s *t*-test were performed to assess differences between categorical and continuous variables, respectively. Univariable and multivariable Cox regression analyses were performed to determine the hazard ratios (HR) with 95% confidence intervals (CI) for DFS and OS, respectively. The relationships between certain independent and dependent variables might be masked by confounding factors, so variables with relaxed *P* < 0.10 in univariate analysis, would be analyzed in multivariable model. Variables with *p* < 0.05 in the multivariate Cox model were considered significant. Kaplan-Meier curves and log-rank tests were performed to evaluate the association between SMI, SMD, VFA, MFA, and OS and DFS.

All parameters (SMA, SMD, VFA, MFA) were independently measured by reviewer A. After four weeks, reviewer B randomly selected 30 patients to evaluate interobserver reproducibility. And these 30 patients were also measured by reviewer A to assess intraobserver variability. Both reviewers were unknown of clinical date. Intraclass correlation coefficient (ICC) was calculated. Reliability interpretation of ICC values were as follows: 0.91–1.0, excellent reliability; 0.75–0.90, good reliability; 0.50–0.75, moderate reliability; 0.01–0.50, none to poor reliability [22]. Correlations between BMI and VFA, MFA, SMD were analyzed using the Spearman’s correlation.

## Results

### Patients’ characteristics

**Fig. 3:**
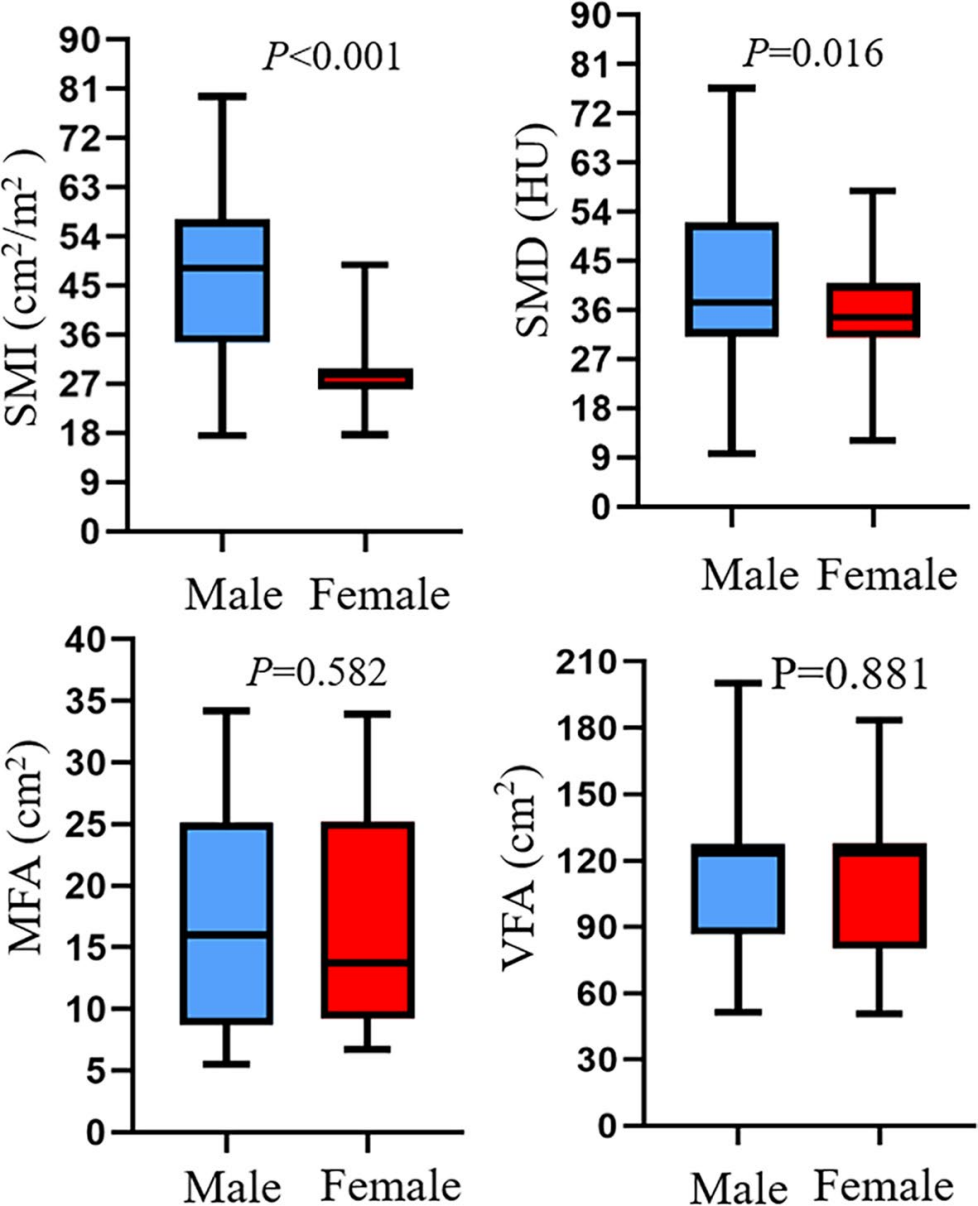
The distributions for SMI, SMD, MFA, VFA in male and female

155 patients (male/female: 99/56, mean age 61.6 ± 12.6 years) with median follow-ups of 65 months (range 7–72 months) were included for the final analysis (Table 1). The distributions for SMA, SMD, VFA and MFA were shown in Fig. 3. The sex-specific median value of SMI was 28.6 cm^2^/m^2^ for females and 48.2 cm^2^/m^2^ for males. The sex-specific median value of SMD was 34.7 HU for females and 37.4 HU for males. The sex-specific median value of VFA was 123.1 HU for females and 123.2 HU for males. The sex-specific median value of MFA was 13.8 cm^2^ for females and 16.0 cm^2^ for males. During the follow-up period, 42 (27.1%) patients had tumor recurrence with 1, 3, 5 year DFS rates of 93, 75, 71%, respectively; 21 (13.5%) patients died with 1, 3, 5 year OS rates of 94, 90, 86%, respectively. Table 2 and Table 3 summarized differences of variables between groups (recurrence vs non-recurrence; death vs non-death). A significantly higher incidence of death was recorded in patients with higher weight (*P* = 0.028) and higher postoperative complications grades (*P* = 0.025).

**Table 1:** Demographic and clinical variables

Characteristics	Mean ± SD (%) [range]
Age (years)	61.6 ± 12.6 [26–87]
Female	56 (36.1)
HTN	41 (26.5)
DM	18 (11.6)
CAD	17 (11.0)
Weight (kg)	59.1 ± 9.3 [40.0–85.0]
Height (m)	1.63 ± 7.5 [1.40–1.80]
BMI (kg/m^2^)	22.1 ± 3.0 [16.1–29.5]
CEA (ng/mL)	61.6 ± 12.6 [26–87]
Length of stay (days)	24.0 ± 8.1 [7–69]
Blood loss (ml)	174.1 ± 407.9 [10–4500]
SMI (cm^2^/m^2^)	40.5 ± 14.4 [17.6–79.7]
SMD (HU)	38.2 ± 13.5 [9.6–76.6]
VFA (cm^2^)	111.1 ± 33.2 [50.6–200.1]
MFA (cm^2^)	17.1 ± 8.9 [5.5–34.2]
Per-op CCRTx	25 (16.1)
Post-op CCRTx	79 (51.0)
TNM stage	
I/II/III/IV	27(17.4)/60(38.7)/68(43.9)/10(5.6)
ASA grade	
1/2/3	63(40.6)/70(45.2)/22(14.2)
Complications	
None/ Minor/ Major	87(56.1)/40(25.8)/28(18.1)

*ASA* American Society of Anesthesiologists, *BMI* body mass index, *CAD* coronary heart disease, *CCRTx* chemoradiation therapy, *CEA* carcinoembryonic antigen, *DM* diabetes mellitus, *HTN* hypertension, *MFA* area of mesorectal fat, *SMD* skeletal muscle radiodensity, *SMI* skeletal muscle index, *TNM* tumor-node-metastasis, *VFA* area of visceral fat

**Table 2:** Comparison of general patient characteristics between groups according to cancer recurrence and survival states

Characteristics	Recurrence			Death		
	Yes	No	*P*	Yes	No	*P*
	*N* = 42	*N* = 113		*N* = 21	*N* = 134	
Age (years)	62.8 ± 12.6	61.1 ± 12.6	0.480	58.7 ±12.3	62.0 ±12.6	0.261
Female	14(33.3)	42(37.2)	0.659	11(52.4)	45(33.6)	0.095
HTN	10(23.8)	31(27.4)	0.649	6(28.6)	35(26.1)	0.813
DM	3(7.1)	15(13.3)	0.437	0(0.0)	18(13.4)	0.156
CAD	7(16.7)	11(9.7)	0.360	2 (9.5)	16(11.9)	1.000
Weight (kg)	59.8 ± 8.6	58.8 ± 9.6	0.582	55.0 ± 9.7	59.7 ± 9.1	0.028*
Height (m)	1.63 ± 6.9	1.63 ± 7.8	0.735	1.61 ± 7.3	1.63 ± 7.5	0.121
BMI (kg/m2)	22.2 ± 2.1	22.1 ± 3.2	0.743	21.1 ± 2.9	22.3 ± 3.0	0.095
CEA (ng/mL)	5.5 ± 9.4	10.0 ± 20.1	0.058	13.4 ± 26.5	8.1 ± 16.2	0.378
Blood loss (ml)	180.7 ± 320.7	171.6 ± 437.2	0.902	135.7 ± 112.0	180.1 ± 436.5	0.645
ASA grade			0.449			0.479
1	19(45.2)	44(38.9)		10(47.6)	53(39.6)	
2	18(42.9)	52(46.0)		7(33.3)	63(47.0)	
3	5(11.9)	17(15.0)		4(19.0)	18(13.4)	
Complications			0.074			0.025*
None	18(42.9)	69(61.1)		7(33.3)	80(59.7)	
Minor	16(38.1)	24(21.2)		6(28.6)	34(25.4)	
Major	8(19.0)	20(17.7)		8(38.1)	20(14.9)	
Length of stay (days)	25.2 ± 10.0	23.5 ± 7.3	0.245	23.1 ± 6.7	24.1 ± 8.3	0.607

*Significant *P* values

*ASA* American Society of Anesthesiologists, *BMI* body mass index, *CAD* coronary heart disease, *CEA* carcinoembryonic antigen, *DM* diabetes mellitus, *HTN* hypertension

**Table 3:** Comparison of pathological findings between groups according to cancer recurrence and survival states

Pathology	Recurrence			Death		
	Yes	No	*P*	Yes	No	*P*
	*N* = 42	*N* = 113		*N* = 21	*N* = 134	
Per-op CCRTx			0.912			0.065
(+)	7(16.7)	18(15.9)		0(0.0)	25((18.7)	
(−)	35(83.3)	95(84.1)		21(100)	109(81.3)	
T stage			0.769			0.783
T1	5(11.9)	9(8.0)		2(9.5)	12(9.0)	
T2	7(16.7)	18(15.2)		3(14.3)	22(16.4)	
T3	15(35.7)	49(43.4)		7(33.3)	57(42.5)	
T4	15(35.7)	37(32.7)		9(42.9)	43(32.1)	
N stage			0.590			0.246
N0	21(50.0)	59(52.2)		9(42.9)	71(53.0)	
N1	12(28.6)	24(21.2)		8(38.1)	28(20.9)	
N2	9(21.4)	30(26.5)		4(19.0)	35(26.1)	
M stage			0.385			0.356
M0	40(95.2)	104(92.0)		18(85.7)	126(94.0)	
M1	2(4.8)	9(8.0)		3(14.3)	8(6.0)	
TNM stage			0.867			0.321
I	8(19.0)	19(16.8)		2(9.5)	25(18.7)	
II	12(28.6)	38(33.6)		6(28.6)	44(32.8)	
III	20(47.6)	48(42.5)		10(47.6)	58(43.3)	
IV	2(4.8)	8(7.1)		3(14.3)	7(5.2)	
Pos-op CCRTx			0.830			0.204
(+)	20(47.6)	56(49.6)		8(38.1)	71(53.0)	
(−)	22(52.4)	57(50.4)		13(61.9)	63(47.0)	

*Significant *P* values

*CCRTx* chemoradiation therapy, *TNM* tumor-node-metastasis

### Factors associated with OS and DFS

Univariate Cox analyses identified four prognostic factors, including BMI (*P* = 0.087), postoperative complications grades (*P* = 0.035), SMI (*P* = 0.042), SMD (*P* = 0.037) for OS, with *P*-values less than 0.10. Among them, SMI (*P* = 0.036), SMD (*P* = 0.022), and postoperative complications grades (*P* = 0.042) remained significant markers for death in multivariable analysis. However, MFA and VFA were not significant prognosis factors for death either in univariable or multivariable analyses (Table 4). In univariable Cox analyses, postoperative complications grades (*P* = 0.076), MFA (*P* = 0.032), SMD (*P* = 0.012) showed significant differences between recurrence and non-recurrence group. After multivariable analysis, MFA (*P* = 0.022), SMD (*P* = 0.011) still reminded statistically significant. However, VFA and SMI were not significant prognosis factors for cancer recurrence either in univariable or multivariable analyses (Table 5).

**Table 4:** Prognostic factors for overall survival (OS), univariate and multivariate analyses

Prognostic factor	Univariate		Multivariate	
	HR (95% CI)	*P*	HR (95% CI)	*P*
Age (years)	0.983(0.952–1.015)	0.301		
Sex	0.505(0.214–1.189)	0.118		
BMI (kg/m^2^)	0.873(0.747–1.020)	0.087*	0.864(0.745–1.002)	0.054
CEA (ng/mL)	1.011(0.995–1.027)	0.191		
TNM stage		0.304		
I	Reference	Reference		
II	1.650(0.333–8.175)	0.540		
III	2.147(0.470–9.801)	0.324		
IV	5.351(0.893–32.044)	0.066		
Complications		0.035*		0.042*
None	Reference	Reference	Reference	Reference
Minor	1.939(0.652–5.770)	0.234	1.825(0.607–5.487)	0.284
Major	3.884(1.408–10.716)	0.009	3.792(1.366–10.525)	0.010
ASA grade		0.490		
1	Reference	Reference		
2	0.605(0.230–1.590)	0.308		
3	1.118(0.351–3.564)	0.851		
Per-op CCRTx	0.037(0.000–4.974)	0.188		
Post-op CCRTx	0.583(0.242–1.407)	0.230		
Low-VFA vs high-VFA	1.328(0.560–3.152)	0.520		
Low-MFA vs high-MFA	0.877(0.372–2.065)	0.764		
Low-SMI vs high-SMI	2.673(1.037–6.890)	0.042*	2.776(1.071–7.196)	0.036*
Low-SMD vs high-SMD	2.745(1.065–7.077)	0.037*	3.058(1.178–7.941)	0.022*

*Significant *P* values

For risk factors with more than 2 categories, the first category was considered as the reference group

*ASA* American Society of Anesthesiologists, *BMI* body mass index, *CCRTx* chemoradiation therapy, *CEA* carcinoembryonic antigen, *MFA* area of mesorectal fat, *SMD* skeletal muscle radiodensity, *SMI* skeletal muscle index, *TNM* tumor-node-metastasis, *VFA* area of visceral fat

**Table 5:** Prognostic factors for disease-free survival (DFS), univariate and multivariate analyses

Prognostic factor	Univariate		Multivariate	
	HR (95% CI)	*P*	HR (95% CI)	*P*
Age (years)	1.008(0.984–1.033)	0.499		
Sex	1.128(0.594–2.144)	0.712		
BMI (kg/m^2^)	1.000(0.905–1.105)	0.998		
CEA (ng/mL)	0.980(0.946–1.015)	0.256		
TNM stage		0.905		
I	Reference	Reference		
II	0.821(0.335–2.007)	0.665		
III	1.073(0.472–2.435)	0.867		
IV	0.932(0.198–4.391)	0.929		
Complications		0.076*		0.074
None	Reference	Reference	Reference	Reference
Minor	2.190(1.116–4.297)	0.023	2.220(1.131–4.359)	0.020
Major	1.574(0.684–3.621)	0.289	1.478(0.641–3.406)	0.359
ASA grade		0.699		
1	Reference	Reference		
2	0.782(0.411–1.491)	0.456		
3	0.731(0.273–1.959)	0.533		
Per-op CCRTx	0.934(0.415–2.104)	0.870		
Post-op CCRTx	1.036(0.565–1.899)	0.909		
Low-VFA vs high-VFA	1.095(0.598–2.007)	0.769		
Low-MFA vs high-MFA	0.502(0.267–0.943)	0.032*	0.478(0.254–0.901)	0.022*
Low-SMI vs high-SMI	1.151(0.628–2.110)	0.649		
Low-SMD vs high-SMD	2.248(1.195–4.228)	0.012*	2.280(1.211–4.291)	0.011*

*Significant *P* values

For risk factors with more than 2 categories, the first category was considered as the reference group

*ASA* American Society of Anesthesiologists, *BMI* body mass index, *CCRTx* chemoradiation therapy, *CEA* carcinoembryonic antigen, *MFA* area of mesorectal fat, *SMD* skeletal muscle radiodensity, *SMI* skeletal muscle index, *TNM* tumor-node-metastasis, *VFA* area of visceral fat

**Fig. 4:**
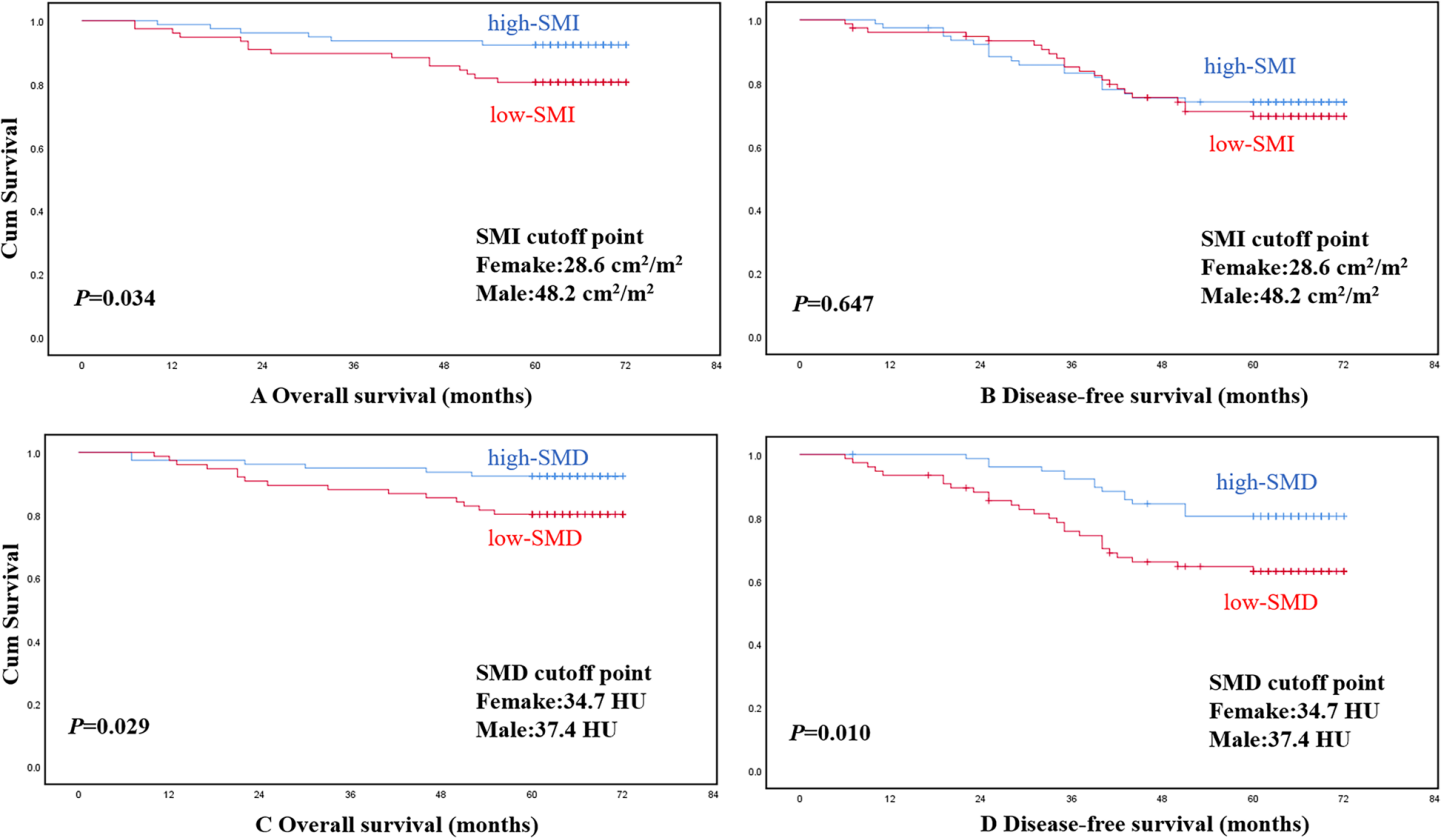
Overall survival or disease-free survival according to SMI and SMD, respectively

**Fig. 5:**
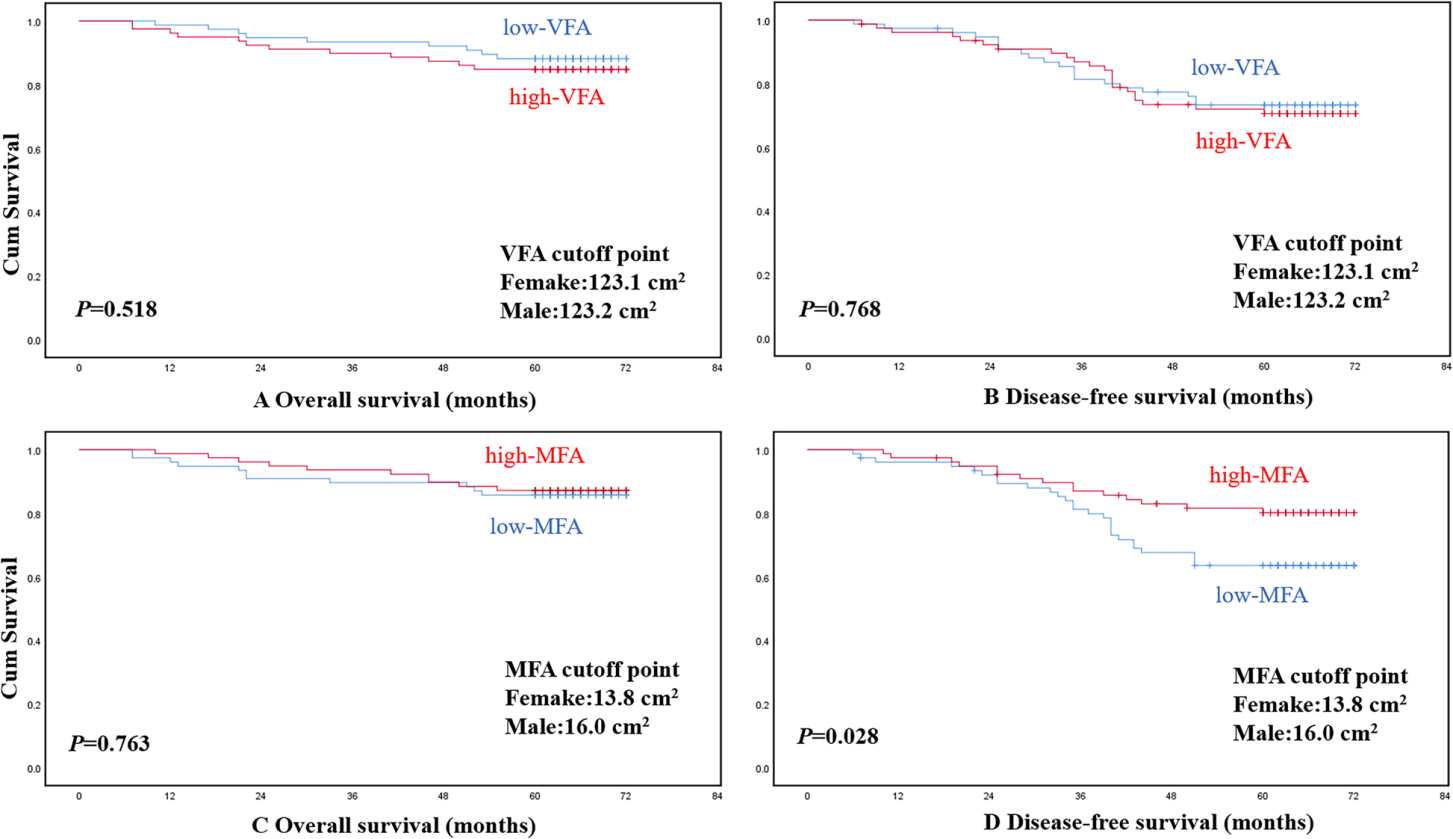
Overall survival or disease-free survival according to VFA and MFA, respectively

Using the Kaplan—Meier method and log-rank test, OS rather than DFS was significantly longer in patients with high-SMI compared to patients with low-SMI (*P* = 0.043). Both OS and DFS were significantly longer in patients with high-SMD compared to patients with low-SMD (*P* = 0.029, *P* = 0.010, respectively) (Fig. 4). DFS rather than OS was significantly longer in patients with high-MFA than patients with low-MFA (*P* = 0.028). No significant differences were found in OS or DFS between high-VFA group and low-VFA group (Fig. 5).

### Inter- and intraobserver reproducibility

Both intraobserver (ICC 0.946, 95CI% 0.890–0.974) and interobserver reproducibility (ICC 0.935, 95CI% 0.870–0.969) were excellent for MFA. Both intraobserver (ICC 0.927, 95CI% 0.853–0.964) and interobserver reproducibility (ICC 0.929, 95CI% 0.856–0.966) were excellent for SMA. Both intraobserver (ICC 0.908, 95CI% 0.818–0.955) and interobserver reproducibility (ICC 0.889, 95CI% 0.780–0.946) were good for SMD.

### Correlation

No correlations were observed between BMI and VFA (*r* = 0.052, *P* = 0.518), and between BMI and MFA (*r* = 0.106, *P* = 0.190), and between BMI and SMD (*r* = − 0.082, *P* = 0.313).

## Discussion

In our study, lower SMI was only a negtive prognostic factor for OS in patients with middle and low RC who received radical surgery, and it had no significant impact on DFS. Lower SMD showed a significant negtive impact both for OS and DFS. VFA failed to show any significant impact both for OS and DFS. MFA was an independent predictor of DFS but not of OS. These results suggested that quantitative evaluation of skeletal muscle mass and adipose tissue distributions at the time of diagnosis is a crucial factor affecting long-term oncologic outcomes.

In accordance with our results, sarcopenia (actually refers to myopenia, the same below) had been reported to be predictive of OS but not of DFS in patients undergoing oncological rectal surgery in many other studies [4, 7]. On the other hand, several lines of evidence showed a significant impact of sarcopenia on OS as well as DFS [23, 24]. In the C SCANS study, sarcopenia was demonstrated to be an independent predictor for a decreased DFS and OS in non-metastatic CRC [23]. In patients undergoing excision of colorectal liver metastases, Vledder et al. discovered that sarcopenia was strongly linked to poorer OS and DFS [24]. In contrast, Nakanishi et al. analyzed 494 patients with stage I–IV CRC，neither OS nor DFS was significantly correlated with sarcopenia [25]. These conflicting results regarding the survival impact of sarcopenia on patients with RC might be partially due to the different ethnicities of study populations. The prevalence of sarcopenia varied considerably according to different ethnicities and cut-off point [26]. Of the 2470 patients included in a American population-based study.

(C SCANS study), 1078 (44%) were classified as having sarcopenia [23]. Of the 494 patients included in a Japanese population-based study (Nakanishi et al.), 298 (60%) had sarcopenia [25]. These studies used the L3-SMI to define sarcopenia with identical cut-off point (38.5 cm^2^/m^2^ for females and 52.4 cm^2^/m^2^ for male).

The loss of muscle mass might be not always observed in cross-sectional area, since CT scan could not differentiate fatty tissue from muscle tissues, decreased muscle mass and increased intramuscular fat accumulation might be regarded as normal SMA when measured by CT. Myosteatosis, referred to inter- and intramuscular fat accumulation, had been reported to reflect muscle strength and quality [16, 17]. Myosteatosis seemed to occur independently of muscle mass depletion and perhaps worked synergistically [18]. Therefore, myosteatosis rather than myopenia might be a better predictor for adverse oncological outcomes in RC patients. Our results revealed that myosteatosis (lower SMD) was a marker for worse OS as well as DFS, while myopenia had no significant impact on DFS. These were consistent with several previous studies that showed a negative impact of myosteatosis on long-term clinical outcomes among CRC patients [19, 27]. The exact physiological mechanism of increased myosteatosis had remained largely unclear, although some theories have been suggested. For example, Malietzis et al. identified a connection between systemic inflammatory response and myosteatosis in patients with CRC, implying that higher neutrophil-to-lymphocyte ratio is an independent risk factor for myosteatosis [28]. Other theories included mitochondrial dysfunctions, leptin signaling defectiveness, neuromuscular changes contributing to decreased regenerative capacity, and the involvement of fibroadipogenic precursor cells [29–32]. Some hypotheses have also been proposed to explain the relationship between myosteatosis and long-term prognosis for cancer patients. Skeletal muscle is the largest site for insulin-dependent glucose storage. Free fatty acids and inter- or intramuscular fat accumulation according to Dresner et al. can cause insulin resistance in human skeletal muscle by inhibiting glucose transport activity [33]. There is mounting evidence that cardiometabolic diseases are related to myosteatosis and insulin resistance, and both have important roles in disease development [34]. The pathogenesis of myosteatosis and its association with prognostic survival in patients with RC should be subjected to further investigation, since the suitable interventions either before or after treatment may enhance these patients’ quality of life.

Obesity was found to be a protective factor for patients with CRC in certain studies, but it was also associated with a higher risk of overall mortality for various diseases in others. This phenomenon is called the obesity paradox [9]. One possible explanation for these inconsistent results was that most studies used BMI as their main criterion for evaluating obesity. However, BMI can’t tell the difference between muscle and fat mass, hardly reflecting adipose tissue distribution [9]. Previous studies reported that exact adipose tissue distribution measured by CT was associated with oncologic outcomes both positively and negatively [35]. Subcutaneous fat tissue (SFT) appeared to be a protective factor against adverse prognosis; on the other hand, visceral fat tissue (VFT) did correlate with increased cancer-related mortality [36]. In our study, BMI, as well as VFA, failed to show any significant impact on long-term outcomes for RC patients. This result might come from different study population selections. Our study included all patients diagnosed with middle and low RC regardless of tumor staging and therapeutic schedule, while others did not [35, 37].

Surgery for middle and low RC is performed within the narrow pelvic cavity and requires precise TME, it is considered to be even more affected by visceral obesity. Smaller mesorectal volume possibly indicates a smaller distance between the mesorectal fascia and tumor, which means that the tumor has a high tendency to invade mesorectal fascia or adjacent organs. Mesorectal fat is a barrier to the spread of tumor locally, and has a protective effect against intra-mesorectal lymph node micrometastases. Therefore larger mesorectal volume could reduce the chance of residual tumor after TME surgery [21, 38]. Dilek et al. also reported that mesorectal volume could be utilized as an independent and new biomarker for predicting pathological response to neoadjuvant chemoradiotherapy in patients with locally advanced RC, and that response-positive individuals had a greater mesorectal volume [39]. Our findings of better DFS with higher MFA might be because TME surgery and preoperative radiotherapy have an overall effect on local cancer control, and survival is mainly determined by distant metastases.

There are several limitations to our study. First, this was a single-center retrospective study with a potential selection bias. 35 cases (18.4%) were excluded from our study due to incomplete follow-up data, severe organ insufficiency, and lack of a preoperative CT scan. Nevertheless, our study still included considerable eligible patients withI- IV tumor stage in the final analyses. Second, myosteatosis was measured by CT in our study, although this widely used imaging tool could not directly assess the location of intramyocellular or extramyocellular fat storage. Still, the radiation attenuation of muscle was highly correlated with direct measurement of muscle lipid content by biopsy and wildly used in myosteatosis-related research [18, 19]. Third, compared with the whole mesorectal volume measured at the level of ischial spine in our study, the amount of fat at the tumor’s level might play a different role for patient outcomes. And this has to be further investigated.

## Conclusions

Quantitative evaluation of skeletal muscle mass and adipose tissue distributions at initial diagnosis were important factors for long-term oncologic outcomes in RC patients. SMD and SMI were independent factors for predicting OS in patients with middle and low rectal cancer who had radical surgery. SMD and MFA were independent factors for predicting DFS in patients with middle and low rectal cancer who had radical surgery.

## Data Availability

The datasets used and/or analysed during the current study are available from the corresponding author on reasonable request.

## Abbreviations

ASA: American Society of Anesthesiologists
BMI: Body mass index
CEA: Carcino-embryonic antigen
CCRTx: Chemoradiation therapy
CT: Computed tomography
DFS: Disease-free survival
MFA: Mesorectal fat area
OS: Overall survival
RC: Rectal cancer
SMD: Skeletal muscle radiodensity
SMI: Skeletal muscle index
TNM: Tumor-node-metastasis
VFA: Visceral fat area
